# 2023: a soil odyssey–HeAted soiL-Monoliths (HAL-Ms) to examine the effect of heat emission from HVDC underground cables on plant growth

**DOI:** 10.1186/s13007-024-01283-3

**Published:** 2024-10-25

**Authors:** Ken Uhlig, Jan Rücknagel, Janna Macholdt

**Affiliations:** Faculty of Natural Sciences III, Department of Agronomy and Organic Farming, Martin-Luther-University, Betty-Heimann-Straße 5, 06120 Halle (Saale), Germany

**Keywords:** Greenhouse experiments, Soil-plant interactions under heat stress, High-voltage direct-current (HVDC) transmission, Heat stress on plants, Underground HVDC cables, Root-zone temperature, Thermotropism, Energy transmission infrastructure impact, Thermal soil effects on crop yield, Heated soil monoliths (HAL-M)

## Abstract

**Background:**

The use of renewable energy for sustainable and climate-neutral electricity production is increasing worldwide. High-voltage direct-current (HVDC) transmission via underground cables helps connect large production sides with consumer regions. In Germany, almost 5,000 km of new power line projects is planned, with an initial start date of 2038 or earlier. During transmission, heat is emitted to the surrounding soil, but the effects of the emitted heat on root growth and yield of the overlying crop plants remain uncertain and must be investigated.

**Results:**

For this purpose, we designed and constructed a low-cost large HeAted soiL-Monolith (HAL-M) model for simulating heat flow within soil with a natural composition and density. We could observe root growth, soil temperature and soil water content over an extended period. We performed a field trial-type experiment involving three-part crop rotation in a greenhouse. We showed that under the simulated conditions, heat emission could reduce the yield and root growth depending on the crop type and soil.

**Conclusions:**

This experimental design could serve as a low-cost, fast and reliable standard for investigating thermal issues related to various soil compositions and types, precipitation regimes and crop plants affected by similar projects. Beyond our research question, the HAL-M technique could serve as a link between pot and field trials with the advantages of both approaches. This method could enrich many research areas with the aim of controlling natural soil and plant conditions.

## Background

From 1987 to 2018, the global primary energy consumption rose from 271 to 580 EJ, with the majority attributable to fossil fuels [1]. Due to the COVID-19 crisis, the demand was reduced to 557 EJ in 2020, primarily because of the 9.3% lower oil consumption [2]. Although this trend is needed (according to BP) to reach the goals of the Paris Agreement for limiting global warming [2], the worldwide total primary energy consumption exceed 2019 levels by 2.8% in 2022 [3]. Therefore, the substitution of fossil fuel will still remain mandatory to achieve these goals. Renewable energy (RE) sources (wind energy, solar energy, hydropower, bioenergy, and geothermal energy) exhibit the highest potential for replacing fossil fuels [4]. In 2021, the annual growth rate reached an all-time high of 15%, surpassing that of all other fuels [5]. Considering these numbers and the need for change, even the implementation of 100% RE systems has been determined feasible in most publications [6], with the highest potential for solar and wind resources [7].

Although the use of RE can negatively affect the environment, such as increased land use intensity [8] and deterioration in avifauna [9] or biodiversity [10], it is still a viable approach for mitigating global warming due to fossil fuel-associated CO_2_ emissions [11–13]. Solar and wind capacity levels have greatly increased over the last three years [3, 5], and 30% of the global electricity generation can already be attributed to RE [14]. However, the above success in RE utilization is associated with a few challenges. In addition, the integration of RE in existing power systems can be difficult [15–17], energy storage capacity is needed [18–21], and transmission is crucial [15, 22, 23].

Within this context, renewable energy resources often occur in isolated areas far from consumption points [24–26]. Hence, newly constructed transmission lines, power grids and interconnectors are needed. There are a vast number of diverse concepts and projects to interconnect different regions worldwide involving multiple supergrids [27, 28] or even a global grid [29]. With the exit of nuclear power in 2023 and extension of the transmission system for transferring power from the north to the south, Germany is already considered to lead the way in achieving a continental supergrid [30–32]. Including RE-rich North Africa, for instance, realizing this supergrid could involve establishing up to 286.260 km grid lines [33].

Although large parts of this grid would comprise sea cables or continental overhead lines, due to local citizen protests regarding environmental concerns, visual disruptions, and expected economic losses, underground cables have been increasingly considered [31, 34]. The German Federal Network Agency (BNetzA) has identified almost 5,000 km of new power line projects (excluding offshore projects), with an initial start in 2038 or earlier as underground cables [35]. It has been predicted that future expansions of power grids worldwide, particularly in dense areas, will also necessitate underground solutions.

In addition to the high-voltage alternating current (HVAC) technique for power transmission, the high-voltage direct current (HVDC) technique has been increasingly employed. To date, HVDC cables are mainly used for connecting offshore wind farms [32], but HVDC power transmission is also used in more than 20 continental transmission lines in China for connecting major RE resources in the west with consumers in the east [26]. Furthermore, the HVDC technique offers advantages in long-distance transmission, integrating RE sources, interconnecting asynchronous networks and providing several technical and commercial benefits [32, 36, 37]. This technique will also be applied in 70% of the earlier mentioned German underground cable grid extension projects [38].

HVDC cables emit heat during operation, and the temperature depends on various factors, e.g., current loading, cross-sectional area of the cable conductor and cable insulation material [39, 40]. While testing a 500-kV cable with the latest isolation material, the temperature on the outside could exceed 50 °C [41].

Furthermore, the surrounding ground can function as a thermal isolator [42]. The thermal conductivity of soil is complex but is mainly determined by the water content, dry density, mineral components and porosity [43, 44].

Even though there are papers considering various technical aspects of cables or the soil temperature [45–47], the effect of heat emission on the yield and root growth of crop plants has not been comprehensively addressed, although this phenomenon is important in regard to compensation claims submitted by farmers fearing a possible yield loss.

Root growth in response to the soil temperature is referred to as thermotropism. Although this subject was introduced more than 100 years ago, in general, it has been underexplored. Currently, depending on the plant species, roots either seek or avoid elevated temperatures [48, 49].

In Germany, there are few expert reports funded by the government or grid operators regarding power lines with a capacity of 380 kV or less. The results showed that the temperature in the upper soil layer (0–40 cm) highly fluctuates by approximately 5 °C [50]. Based on this research, the German Federal Network Agency officially invalidated possible yield losses in 2017 [51], although a report of the Federal Ministry for Economic Affairs and Energy (BMWi) rated the impact as not broadly evaluated [52]. Another report erroneously indicated that only the upper 50 cm of soil is relevant for crops [53].

Whereas the majority of the root biomass of common crop plants occurs within the top 50 cm of soil [54], deep rooting (to 2 m or deeper) for robust growth is common [55] and will become increasingly important with increasing climate challenges. Regardless, the roots of overlying plants will grow in the area thermally affected by HVDC cables, which has raised concerns among farmers.

To examine the possible effects of HVDC underground cables on root growth and crop yield, a long-term field experiment involving an actual 525-kV line would constitute the best approach. However, this is a very expensive, time-consuming, and weather-dependent approach, which is difficult to accomplish due to the high voltage. In situ root observation through a minirhizotron would also be limited.

Therefore, we evaluated different crop experiments in the literature to obtain an ideal solution. Sophisticated semifield experiments and rhizo-lysimetric approaches were discarded due to the limited budget [56, 57]. The target was to establish a suitable vessel experiment for a greenhouse with the advantage of controlling precipitation, climate and soil‒crop combinations. Normal planting pots, containers and standardized experimental pots such as ‘Mitscherlich’ vessels are unsuitable for most crop plant experiments due to the lack of an appropriate plant‒soil ratio, especially with the aim of maintaining growth until maturity [58]. Moreover, a representative soil profile is needed to consider heat transmission through the soil. We found a broad variety of alternatives in different scientific fields including large-scale containers [59], RhizoTubes [60] and soil columns [61, 62]. In addition, we exclusively focused on thermal effects and simulated heat emission.

Considering all the above points, our goals were to construct a low-cost large vessel prototype (i) to control the temperature at a depth of 1.40 m for simulating heat flows originating from an HVDC cable, (ii) with a natural soil composition and density, that can be used (iii) to monitor the soil temperature, soil water content and root growth (iv) and that can be installed in a greenhouse to control precipitation and climate (v). This approach could facilitate significant advancements in field experiments, with faster and more comprehensive results, reliable climate and precipitation control and lower investment. This experimental setup should focus on examining potential heat flow effects in Saxony-Anhalt resulting from the planned SuedOstLink project, which involves a 525-kV HVDC underground power line traversing this federal state in the center of Germany [35].

## Methods

The experiment was conducted in a greenhouse at the university field trial station Halle/Kühnfeld from late 2019 to late 2022. Large vessels were constructed to evaluate the effect of heat emission (HEAT) on two soil types (LOESS and SAND), thereby considering three precipitation levels (DRY, MID, and WET) and three crop plants (spring barley, sugar beet and spring wheat), each with two replicates ( Table 1). The plants were cultivated in three successive growth phases (GPs) simulating crop rotation. Our aim was to construct low-cost large vessels with natural soil conditions to simulate heat emission at a depth of 1.40 m and to evaluate the effect of heat flow on crop rotation in a controlled environment in less time than field trials. The soil temperature, soil water content, root intensity and yield were compared between HEAT and control (CTRL).

**Table 1:** **Overview of factors, factor levels and replicates**

	24 × HeAted soiL Monoliths (HAL-Ms)											
SOIL	12 × LOESS						12 × SAND					
SOILTEMP	6 × CTRL			6 × HEAT			6 × CTRL			6 × HEAT		
RAIN	2 × DRY	2 × MID	2 × WET	2 × DRY	2 × MID	2 × WET	2 × DRY	2 × MID	2 × WET	2 × DRY	2 × MID	2 × WET

### Construction of the HeAted SoiL-Monoliths (HAL-Ms)

**Fig. 1:**
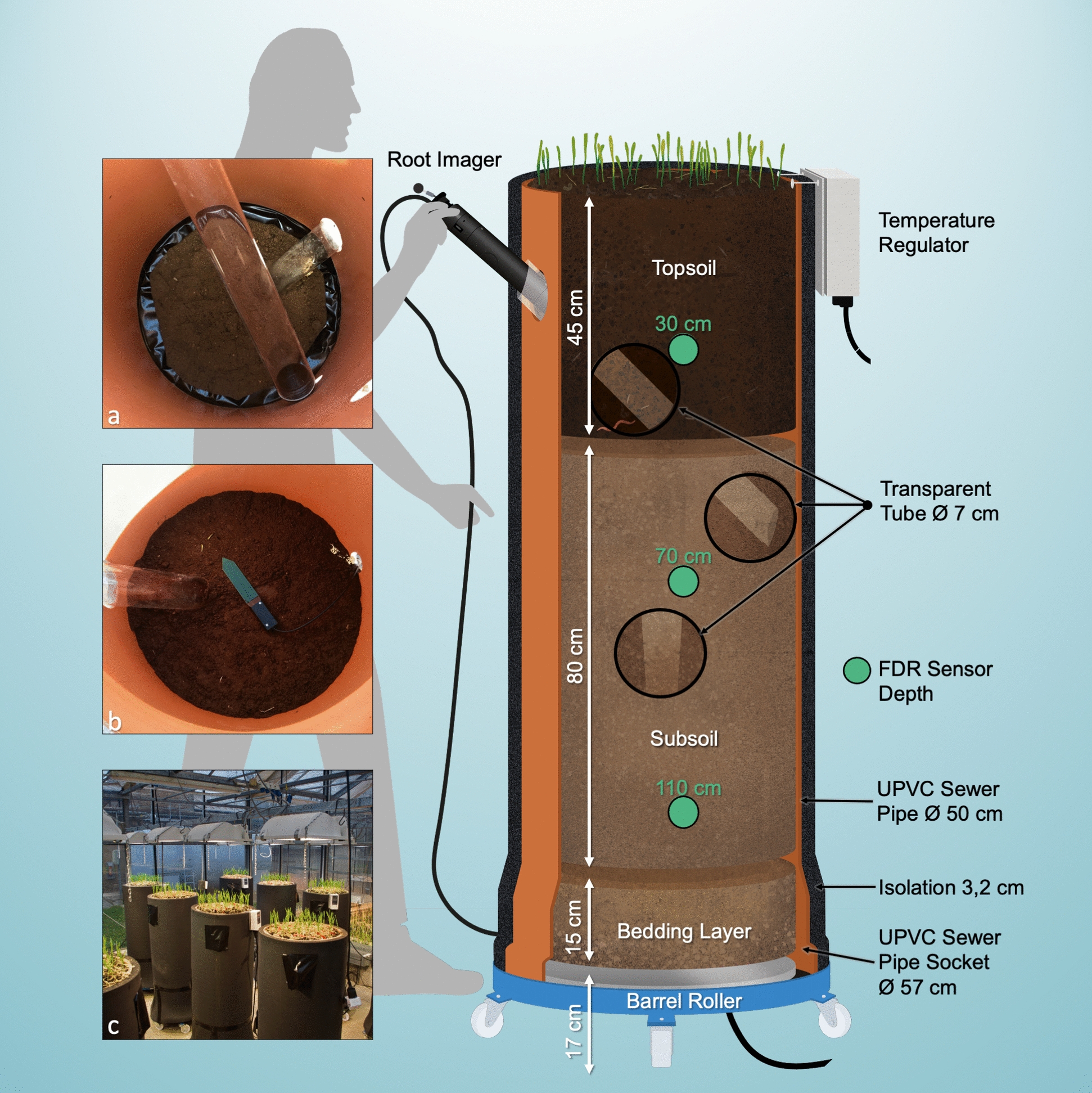
**Construction of the** **HeAted soiL Monoliths (HAL-Ms). a** Subsoil with black duct tape as a root barrier and two transparent tubes offset by 90°; **b** topsoil with an FDR sensor; and **c** finished HAL-Ms in the greenhouse; UPVC = Unplasticized polyvinyl chloride

Twenty-four HAL-Ms with a height of 1.56 m and an approximate diameter of 0.56 m were built for this experimental setup. Each contained approximately 400 kg of soil, with a planting area of 0.177 m^2^. The foundation of the twelve HAL-Ms was a barrel roller (HBR10, Hillesheim), equipped with a built-in heater and six polymer steering rollers (Fig. 1). The interior diameter was 0.61 m, and the height was 0.175 m. The aluminum thermal element exhibited a diameter of 0.51 m. Through a capacity of 1200 W, the heater could reach a temperature of 100 °C, controlled by an external temperature regulator (HT63, Hillesheim) based on proportional–integral–derivative (PID) control. With additional time switches, different day and night temperatures were possible. The other twelve HAL-Ms served as the control group, with identically constructed barrel rollers but with a solid metal plate instead of the implemented heating element.

Unplasticized polyvinyl chloride (UPVC) sewer pipes with a diameter of 50 cm (KG 500 SN 4) were cut to a length of 1.40 m, placed vertically on the socket side on the barrel roller without a seal to ensure the drainage of excess water through the approx. 0.5 cm gap. The sewer pipes were isolated with flexible 32-mm thick elastomeric synthetic rubber foam (Pipe isolation, Armaflex XG 32).

Two transparent root observation tubes (Ø 70 mm) were installed at different heights. The tubes were arranged diagonally to the pipe wall (at an angle of 45°) and offset horizontally to cover a larger root space and to avoid root growth effects resulting from the upper tube (Fig. 1a). Thus, root growth could be observed by the CI-600 In Situ Root Imager (CID Bio-Science) within the ranges of 22.5–53.0 cm and 71.0–101.5 cm beneath the top edge [63–65]. The tops of both transparent tubes were capped and covered to prevent exposure to light (Fig. 1c).

To monitor the soil temperature and moisture, three horizontally aligned frequency domain reflectometry measuring probes (SMT 100, TRUEBNER) were installed at depths of 0.30, 0.70 and 1.10 m. Hence, holes for the cables were drilled and sealed after installation (Fig. 1b). Similarly to other field experiments, we used the manufacturer's calibration instead of a soil-specific calibration because we were more interested in the dynamics of soil moisture, which meant to us the changes and fluctuations in the amount of water held in the soil over time, influenced by the factors SOIL, RAIN and plant growth, rather than its absolute values [66]. The 24 HAL-Ms were randomized and placed in two greenhouse compartments, separated by soil type, and not rotated in the experiment.

### Soil properties

The constructed columns were filled with natural topsoil and subsoil extracted from two sites near Bernburg and Merseburg (Germany, Saxony-Anhalt), representing different regional soil types, i.e., LOESS and SAND. The corresponding soil texture details are listed in Table 2. The two soils were filled in 5-cm layers and manually compacted to obtain the target dry bulk density (BD_target_), which is the observed bulk density obtained from different publications for the considered soil types in this region [67–70]. The top and subsoil layers exhibited thicknesses of 45 and 80 cm, respectively. To meet the given underground cable construction requirements, a 15-cm bedding layer with a given proctor density [71] was installed in advance. During filling, 250-cm^3^ soil cores (Ø 7.2 cm, 6.0 cm high) were collected from different layers for analyzing the field capacity (FC), permanent wilting point (PWP) and bulk density (BD). The soil cores were drained for seven days in sandboxes with a hanging water column to a matric potential of -6 kPa, corresponding to an FC pF value of 1.8 [72]. The PWP (pF 4.2) was estimated by the water content at a soil matric potential of -1.5 MPa [73]. The soil samples saturated hydraulic conductivity (K_S_, cm d^−1^) was measured using a stationary system with a flow duration of 4 h, as described by Klute and Dirksen [74]). The volume of the samples was 250 cm^3^ and their height was 6 cm. To determine the bulk density, the particle density was measured with a helium pycnometer (Ultrapyc 5000, ANTON PAAR). FC_KA5_ refers to a similar texture class mentioned in the German manual of soil mapping KA5 (5th Ed) [75]. To avoid preferential root growth along the inner surface of the pipe, duct tape was affixed in an L shape at 15-cm intervals to lead the roots back to the center (Fig. 1 a). In total, we constructed six heated and six control HAL-Ms for LOESS and SAND each. Soil damage compaction due to the installation and electromagnetic fields of HVDC lines [76] were not considered in this setup. Heat dispersion in soil was only analyzed from a simple perspective.

**Table 2:** **Properties and nutrients of the soil used in the experiments**

	Soil depth	m	Texture %		Texture class(IUSS)	Organic matter %	pH	Nutrients (mg per 100 g)			N_min_ kg ha^-1^	BD g cm^−3^	BD_target_ g cm^−3^	FC vol%	FC_KA5_ vol %	PWP vol %	k_S_ cm d^−1^
			Sand	Clay				P	K	Mg							
LOESS	Topsoil	0–0.45	10	7	Silt	1.28	7.0	3.3	35.7	12.9	201	1.22	1.22	41.7	43.0	15.2	26.6
	Subsoil	0.45–1.25	10	12	Silt loam	0.29	7.6	1.5	4.1	10.1	16	1.40	1.42	42.3	37.0	13.3	9.9
	Bedding	1.25–1.40	15	6								1.58	2.03				
SAND	Topsoil	0–0.45	35	23	Loam	1.86	6.2	4.6	2.9	11.9	36	1.45	1.45	31.7	34.0	15.0	15.7
	Subsoil	0.45–1.25	92	5	Sand	0.35	7.7	1.1	1.6	4.8	12	1.63	1.60	16.5	18.0	7.0	99.7
	Bedding	1.25–1.40	15	6								1.58	2.03				

*Texture, organic matter and nutrients determined by Eurofins Agraranalytik Deutschland GmbH, Jena, Germany. The dry bulk density (BD), field capacity (FC), permanent wilting point (PWP) and saturated hydraulic conductivity (k*_*S*_*) were determined from soil cores collected during construction, representing mean values, with n* = *11 for LOESS and n* = *5 for SAND. BD*_*target*_* (targeted dry bulk density) values were retrieved from different publications (*^*d*^*Altermann* et al.*, 2005; *^*e*^*Kaufmann* et al.*, 2010; *^*b*^*Rücknagel* et al.*, 2017; *^*c*^*Schlüter* et al.*, 2018). FC*_*KA5*_* values were used as reference values for similar texture classes from KA5 *[75]

### Simulated heat emission and water regime

The two investigated factors were SOILTEMP and RAIN (precipitation). Regarding SOILTEMP, half of the HAL-Ms with LOESS and half of those with SAND were heated to a constant temperature of 50 °C (HEAT), while the other half served as the control group (CTRL). During the initiation phase of the setup, a temperature of 40 °C was applied, changing to a day–night cycle on 2 December 2019, with temperatures of 60° C maintained for 16 h and 40° C maintained for 8 h. Finally, the temperature of the heating element was set to 50° C on 12 January 2020 for the remaining experimental time.

Regarding RAIN, three levels were calculated based on data from 1988 to 2018 provided by the German Meteorological Service (DWD). Weather stations in Magdeburg, Bernburg, Leipzig/Halle Airport and Jena, which are located along the planned HVDC line (SuedOstLink), were considered. The first quartile of the dataset, with a mean annual precipitation of 407 mm, was considered as dry (DRY) year, the second and third quartiles, with a mean of 528 mm, were jointly considered as moderate (MID) year, and the fourth quartile, with a mean of 679 mm, was considered as wet (WET) year. Furthermore, the monthly precipitation was calculated based on the annual distribution of rainfall. Precipitation was applied in varying quantities without a specific schedule within the matching month to the BBCH [77] stages. Normal winter precipitation was applied between the growth phases. Precipitation was manually applied during the trial with a graduated beaker. Because we observed slightly faster plant growth at the early stages of GP1, we divided the monthly watering amount over 22 days instead of 30 days. This was also necessary during the later culture phases.

In the region considered for the experiment, the natural soil water content is generally replenished in autumn and winter. Since the soils for the HAL-Ms were removed from field in summer, replenishment was performed artificially before the trial. To avoid waterlogging in the HAL-Ms, the connection between the sewer pipe and barrel roller was not sealed. The measured field capacity was comparable with the literature data (Table 2). At the beginning of the experiment, an average volumetric water content of approximately 70–80% of the FC was achieved, which is considered the optimum range in this region.

### Growing conditions and plant material

The trial was conducted in a greenhouse with the climatic conditions regulated for all crop plants according to their BBCH stages and associated conditions if necessary as following with night / day temperatures and photoperiod in h: (i) germination: 5 °C / 10 °C with 10 h (ii) leaf development and tillering 10 °C / 15 °C with 12 h (iii) stem elongation and booting: 15 °C / 15 °C with 14 h (vi) ear, flower, fruit development: 15 °C / 20 °C with 16 h ripening: 18 °C / 22 °C with 16 h. The light intensity was continuously adjusted to approximately 400 (± 30) μmol m ^−2^ s ^−1^. To avoid the need for vernalization, spring barley (*Hordeum vulgare*) GP1, sugar beet (*Beta vulgaris*) GP2 and spring wheat (*Triticum aestivum*) GP3 were cultivated under crop rotation representing three of the most common crops in the reference region.

A larger amount of spring barley variety KWS Irina was seeded on 27 November 2019, the number of plants was reduced to 52 per HAL-M (290 seeds m^−2^) after emergence, after which the plants were harvested on 17 April 2020. Regarding GP2, nine sugar beet seeds of the Strauss variety were seeded in a circular manner on 08.09.20, and the number of plants was similarly reduced after emergence to three plants per HAL-M (17 seeds m^−2^) and harvested on 19 January 2021. Regarding GP3, spring wheat variety Lennox (facultative wheat) was seeded on 19 February 2021, and after emergence, the number of plants was reduced to 58 plants per HAL-M (330 seeds m^−2^). Harvesting was performed on 16 June 2021. The nutrient content in the soil was determined by Eurofins Agraranalytik (Eurofins Agraranalytik GmbH, Jena, Germany) before the start of crop rotation. Fertilizers, including calcium ammonium nitrate (N) and potassium oxide (K), were applied in accordance with general practice via a nutrient budget before each crop rotation (GP1 LOESS: 0 kg N ha^−1^; SAND: 90 kg N ha^−1^ and 250 kg K ha^−1^; GP2 LOESS: 70 kg N ha^−1^; SAND: 75 kg N ha^−1^; and GP3 LOESS: 77 kg N ha^−1^; SAND: 82 kg N ha^−1^). Plant protection was achieved with beneficial insects *Aphidoletes aphidimyza, Chrysoperla carnea* and *Trichogramma spec.* if necessary. The incidence of fungal or bacterial disease, if any, was very low, rendering treatment unnecessary. Harvest followed after complete crop maturity. Yield and crop residues were separately harvested and dried at 105 °C for 48 h. After weighing, the crop residues conformed with the field principle incorporated in the corresponding HAL-Ms before the start of the next growth phase.

### Root growth observation and analysis

Root images were obtained with a CI-600 In Situ Root Imager (CID Bio-Science), usually seven to eight times per crop, 40 to 110 days after sowing (DAS). Four images per HAL-M were obtained, covering depths ranging from 22.5–53.0 cm and 71.0–101.5 cm. To determine the root intensity, all the images were aligned and superimposed on a counting grid (1 × 1 cm), with a total grid line of 8.14 m [57, 78]. Image editing was performed in easyHDR version 3.14.1 (for image registration) and free software GIMP version 2.10.14, including the BIMP plugin (for batch processing). The brightness and saturation were also adjusted. The root intersections were manually counted under constant comparison of predecessor images. Thus, distinguishing root residue from earlier crops and recognizing roots, especially in the LOESS substrate, could be improved.

### Data analysis

Soil temperature and water content data were collected with a TrueHub datalogger (TRUEBNER GmbH) and further processed with RStudio (version 1.3.1056). Yield and root data were statistically analyzed in Prism 10 (version 10.0.0) using two-way ANOVA (SOILTEMP*RAIN) and Tukey’s multiple comparison test (95% probability level). Regarding the root growth time series, the maximum root intensity was statistically analyzed for each depth interval via two-way ANOVA (SOILTEMP*RAIN).

## Results

### Performance of the HeAted SoiL Monoliths (HAL-Ms)

**Fig. 2:**
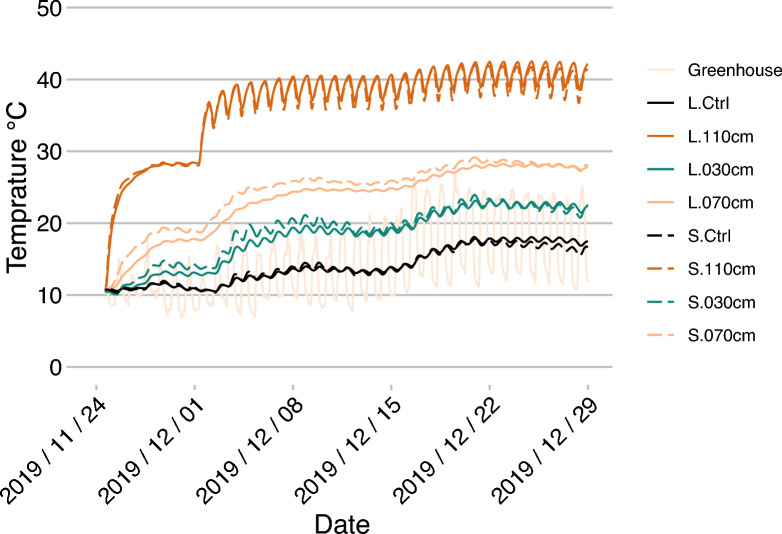
**Soil temperature at the beginning of the study **(40 °C) until the day–night cycle yielded a value of 60/40 °C. Hourly values of the greenhouse ambient temperature (Greenhouse); mean temperature of the CTRL HAL-Ms was calculated from all depths (30, 70, and 110 cm) for the LOESS and SAND treatments (L.Ctrl and S.Ctrl, respectively) as there was no temperature gradient present; and temperature of the HEAT HAL-Ms for the LOESS and SAND treatments at depths of 30, 70 and 110 cm separately

**Fig. 3:**
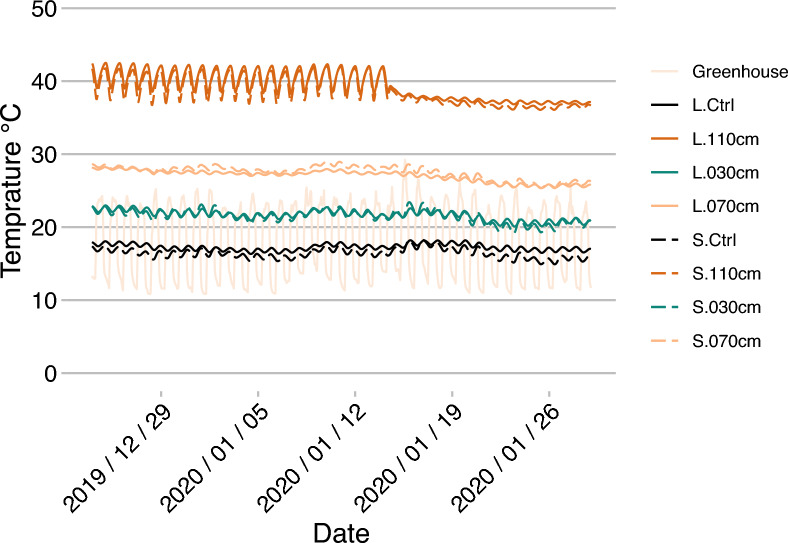
**Soil temperature during the shift from the day–night cycle** (with a temperature of 60/40 °C) to a constant value of 50 °C. Hourly values of the greenhouse ambient temperature (Greenhouse); mean temperature of the CTRL HAL-Ms was calculated from all depths (30, 70, and 110 cm) for the LOESS and SAND treatments (L.Ctrl and S.Ctrl, respectively) as there was no temperature gradient present; and temperature of the HEAT HAL-Ms for the LOESS and SAND treatments at depths of 30, 70 and 110 cm separately

The temperature regime in the heated HAL-Ms was effective and resulted in the expected temperature profiles at the different soil depths. To evaluate the response of the system to different temperature inputs, we started in the first week at 40 °C, as shown in Fig. 2. Within three days, the set temperature notably increased to approximately 28 °C at a depth of 110 cm. With a certain delay, the temperature also increased at 30 and 70 cm but was slightly higher under the SAND treatment. On 2 December 2019, we changed to a day‒night cycle, comprising temperatures of 60 °C for 16 h and 40 °C for 8 h. This led to the temperature fluctuating between 35 and 40 °C at 110 cm, with a value of approximately 40 °C after the greenhouse temperature was adjusted on 19 December (Fig. 3). Due to the adjusted project specifications, the day‒night cycle was discontinued from 15 January 2020, after which the temperature was adjusted to a steady value of 50 °C for the remaining time. This led to a lower energy input and, therefore, lower temperatures.

**Fig. 4:**
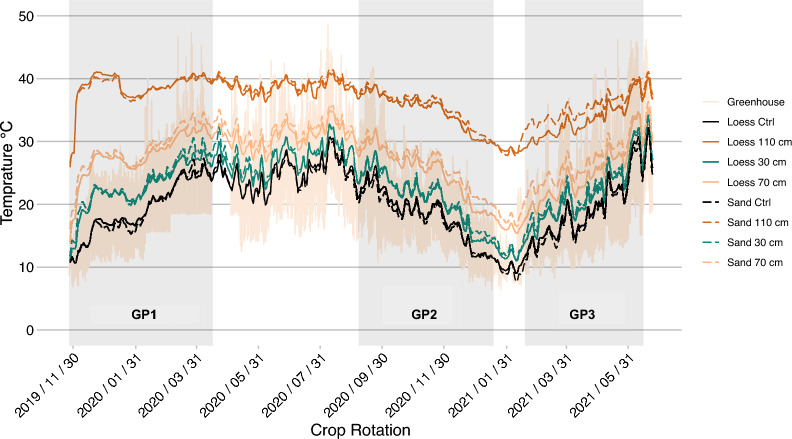
**Daily averages over the whole crop rotation. **With the greenhouse minimum and maximum ambient temperatures (Greenhouse); mean temperature of the CTRL HAL-Ms was calculated from all depths (30, 70, and 110 cm) for the LOESS and SAND treatments (L.Ctrl and S.Ctrl, respectively) as there was no temperature gradient present; and temperature of the HEAT HAL-Ms for the LOESS and SAND treatments at depths of 30, 70 and 110 cm separately. The gray shaded areas denote the growth phases of GP1 spring barley, GP2 sugar beet and GP3 spring wheat

Figure 4 shows the daily mean temperature for the different soils and depths from the start of November 2019 until the end of the experiment in June 2021. The greenhouse ambient temperature is shaded in brown in the background, showing the daily minimum and maximum values. The gray shaded areas denote the growth phases GP1, GP2 and GP3.

During the different growth phases, the soil temperature in the HAL-M’s exhibited a cyclic increase or decrease comparable to the natural soil temperature change at a depth of approximately 1 m (DWD). In general, there was a clear indication that the greenhouse environment influences the soil temperatures in all HAL-Ms. Hence, under the influence of the warm late summer days of 2020, GP2 started with high soil temperatures, which slowly decrease toward the minimum temperature in January 2021. This resulted in an inverted temperature sequence for the greenhouse ambient conditions relative to that of GP1 and GP3. Between GP2 and GP3, the controlled temperature in the greenhouse was reduced to achieve similar starting conditions for soil temperature as at the beginning of GP1. The average ambient temperatures during the growth phases GP1, GP2 and GP3 were 20.5, 19.2 and 19.5 °C, respectively. Short-term peaks in the ambient temperature above 40 °C, as observed during the late GP1 and GP3 and early GP2 growth phases, occurred, especially under weather conditions with abundant sunshine and high wind speeds, due to the safety protocols of automated greenhouse control.

To compensate for the higher ambient temperature due to warm weather conditions, we decided to omit the night temperature setting of 18 °C at the later stages of GP2 and GP3 to allow the HAL-M to cool at night. In general, the temperature of the soil in the control groups followed the daily mean temperature (Fig. 4).

The soil type imposed a minor effect on the temperature in the heated HAL-Ms. At the 110-cm depth, the average differences between the LOESS and SAND treatments for GP1, GP2 and GP3 were 0.3, 0.7, and 2.0 °C, respectively, at the 70-cm depth, the values were 1.1, 1.3, and 2.1 °C, respectively, and at the 30-cm depth, the values were 0.6, 0.4 and 0.7 °C, respectively. There was a notably larger difference during the GP3 phase, indicating slightly higher temperatures under the SAND treatment. This started shortly before GP3 and disappeared at the end.

### Water regimes

**Fig. 5:**
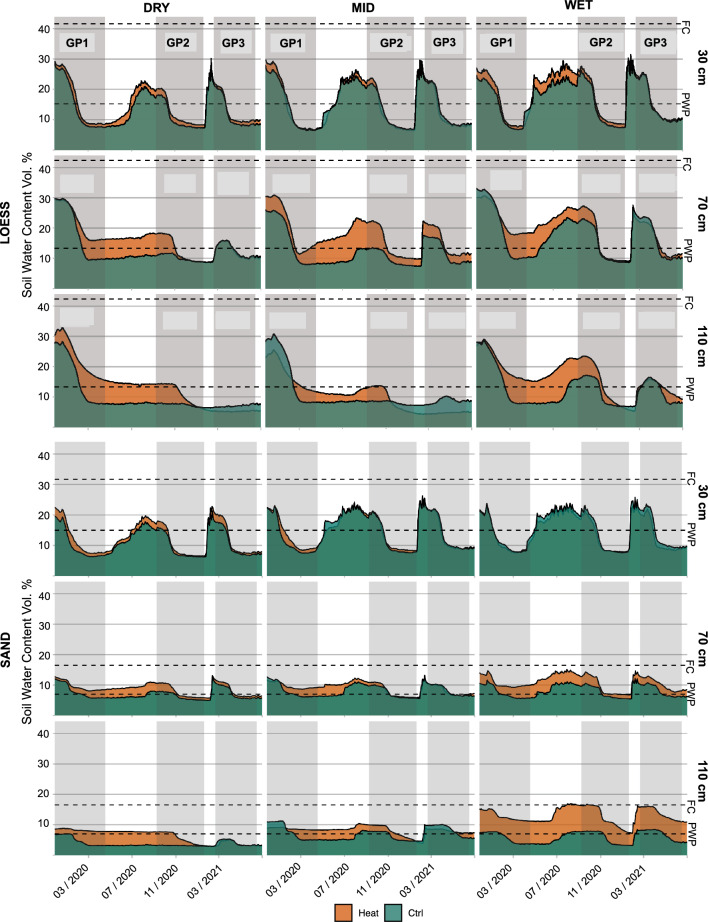
**Volumetric soil water content.** The HEAT and CTRL HAL-Ms were sorted by the level of RAIN, with DRY, MID, and WET conditions and soil depths of 30, 70 and 110 cm for LOESS (L) and SAND (S). The permanent wilting point (PWP) and field capacity (FC) of the soils were determined in the laboratory

Figure 5 llustrates the volumetric soil water content for the different precipitation levels and soil depths under the LOESS and SAND treatments. The gray shaded areas denote the growth phases of GP1, GP2 and GP3. The dashed lines denote the FC and PWP determined in the laboratory. FC in the HAL-Ms was not achieved under any circumstances, while the PWP was slightly lower in some cases. However, the starting values of the soil water content were consistent within the LOESS or SAND treatment, with increasing differences over time due to the precipitation levels.

While the topsoil at the 30-cm depth generally reached its initial values before GP1 and GP2, there was a water lag in the subsoil at 70 and 110 cm.

The DRY level of precipitation under the LOESS treatment was not sufficient to increase the water content, and the MID level was only able to do so at a depth of 70 cm. The WET level could increase the water content, with a delay between 70 and 110 cm, indicating that water naturally flowed from top to bottom. The results were similar for the SAND treatment, albeit less pronounced. Due to the lower FC value under the SAND treatment, the amount of water provided between the GPs was in most cases enough to reach the initial soil water content, but not under the LOESS treatment.

At the middle of each GP, the water content started to decrease due to plant water use. However, there was a notable difference during GP1 between HEAT and CTRL (Fig. 5). Especially in the subsoil of the LOESS treatment, the water content in the heated HAL-Ms decreased less than that in the control group and remained higher until the middle of GP2. From that point, the soil water content levels again reached equilibrium. This effect did not occur during GP3 and was generally less pronounced under the SAND treatment.

### Root growth observation

**Fig. 6:**
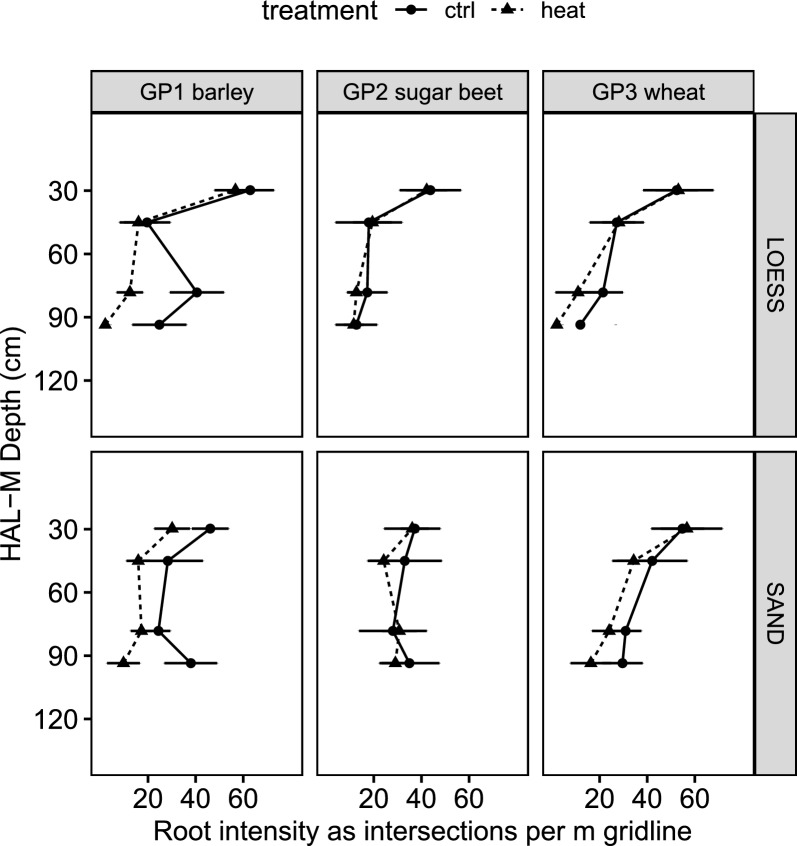
**Maximum observed root intensity for the main factor, SOILTEMP.** The dashed line denotes CTRL, and the solid line denotes HEAT. The error bars indicate the SD values. The observed soil depths are 22–53 cm and 71–101 cm

**Fig. 7:**
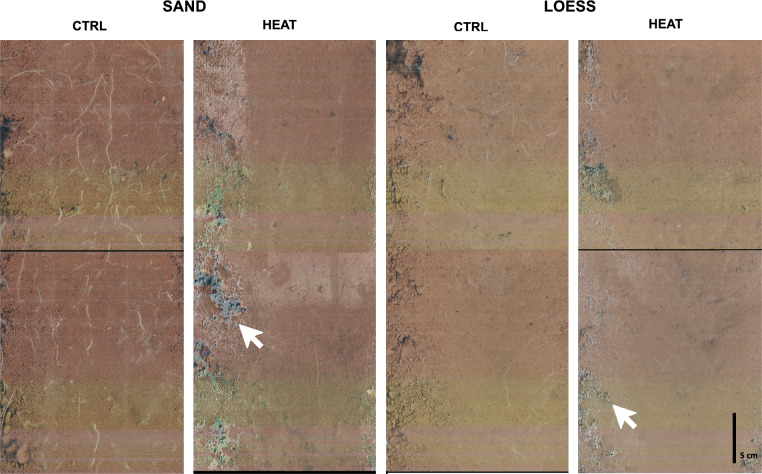
**Images of root growth under heat emission conditions.** Growth under the LOESS and SAND treatments at HAL-M soil depths from 71–101 cm for spring barley 56 days after sowing. The white arrows indicate moisture condensation on the outside of the rhizotron tubes of the heated HAL-Ms

In Fig. 6, the maximum observed root intensity of the three crops under the LOESS and SAND treatments for the main factor SOILTEMP is depicted. Corresponding P values, including factor RAIN and possible interactions, are listed in Table 3. The root intensity during GP1 under simulated heat emission was significantly reduced at soil depths from 71.0 to 101.5 cm under the LOESSS and SAND treatments. Beyond this depth, there was a lower root intensity under the SAND treatment from 22.5–37.5 cm. The three precipitation levels DRY, MID and WET did not affect root growth. In contrast to GP1, HEAT did not affect the intensity of sugar beet roots during GP2. RAIN yielded differences under the LOESS treatment at depths from 22.5–37.5 cm and under the SAND treatment at depths from 86.0–101.5 cm, with interaction between SOILTEMP and RAIN. During GP3, the root intensity at depths from 71.0–101.5 cm under the LOESS treatment was reduced under the influence of HEAT, while precipitation also yielded an impact, with interaction at depths from 86.0–101.5 cm. There was a notable tendency for reduced root growth due to heat emission under the SAND treatment (p value = 0.0585). In addition to the differences in the root intensity during GP1, it could be observed that roots under the influence of HEAT exhibited a yellowish/brownish color relative to the CTRL group, with a vivid white color (Fig. 7). This difference was less obvious under the LOESS treatment than under the SAND treatment because roots were harder to recognize. Even if no significant difference in the root intensity between HEAT and CTRL could be detected during GP2 or GP3, root discoloration was more likely to occur under the influence of HEAT. The white arrows indicate water condensation on the outside of the transparent tubes of the heated vessels (Fig. 7).

**Table 3:** **Root growth statistics**

P values								
HAL-M soil depth in cm	22.5—37.5		37.5—53.0		71.0—86.0		86.0—101.5	
	LOESS	SAND	LOESS	SAND	LOESS	SAND	LOESS	SAND
GP1 Spring barley								
SOILTEMP	0.3460	**0.0215**	0.4105	0.1050	**0.00354**	**0.0481**	**0.0057**	**0.0005**
RAIN	0.7970	0.8787	0.0719	0.5850	0.66297	0.3766	0.7751	0.5864
SOILTEMP*RAIN	0.5010	0.6552	0.5185	0.3740	0.82898	0.7047	0.6107	0.0682
GP2 Sugar beet								
SOILTEMP	0.7039	0.8680	0.7799	0.2700	0.2260	0.6790	0.7430	0.0850
RAIN	**0.0291**	0.5680	0.0613	0.3560	0.2530	0.3850	0.2030	**0.0174**
SOILTEMP*RAIN	0.0875	0.8050	0.6219	0.8650	0.3150	0.3190	0.1920	**0.0201**
GP3 Spring Wheat								
SOILTEMP	0.9400	0.8060	0.8830	0.3780	**0.00969**	0.1440	**0.0084**	0.0585
RAIN	0.5910	0.7030	0.3110	0.7300	**0.00768**	0.3700	**0.0048**	0.5360
SOILTEMP*RAIN	0.9670	0.2850	0.9590	0.6940	0.23023	0.6270	**0.0096**	0.9988

P values of two-way ANOVA (SOILTEMP*RAIN) for the root intensity (n = *2) for growth phases (GP) 1-3, with the bold values indicating p* < *0.05*

### Crop rotation in the greenhouse

The summer crop rotation was successfully cultivated under greenhouse conditions in roughly one and a half years and therefore swifter than possible with field trials. Our developed heated soil monoliths (HAL-Ms) exhibited adequate dimensions; thus, the crops experienced all BBCH stages from sprouting to maturity and were successfully harvested. The yields of the control HAL-Ms were comparable to those under field conditions in Saxony-Anhalt (State Statistical Office of Saxony-Anhalt).

Table 4 provides the mean yields under crop rotation for the different factors SOILTEMP and RAIN, as well as p values. The yield of spring barley during GP1 under the SAND treatment was significantly reduced under the influence of HEAT (423.6 to 261.9 g m^−2^). RAIN also exerted a significant impact on the yield, with higher values under WET than under MID and the lowest yield under DRY. Moreover, HEAT reduced the yield under the LOESS treatment (without statistical certainty).

**Table 4:** **Yield and p value for the growth phases of GP1-GP3**

			Yield g m^−2^			
	GP1 Spring Barley		GP2 Sugar Beet		GP3 Spring Wheat	
	LOESS	SAND	LOESS	SAND	LOESS	SAND
CTRL (n = 6)	382.6 (56.9)	423.6 (15.4)	5609.0 (127.7)	5972.0 (294.0)	314.1 (14.8)	313.3 (20.9)
HEAT (n = 6)	258.8 (56.9)	261.9 (15.4)	6608.0 (127.7)	6055.0 (294.0)	292.4 (14.8)	293.7 (20.9)
DRY (n = 4)	265.3 (69.7)	281.6 (18.9)	4023.0 (156.4)	3848.0 (360.1)	181.0 (16.2)	199.8 (25.6)
MID (n = 4)	283.2 (69.7)	333.2 (18.9)	5824.0 (156.4)	5872.0 (360.1)	304.3 (18.2)	316.3 (25.6)
WET (n = 4)	413.8 (69.7)	413.5 (18.9)	8479.0 (156.4)	8320.0 (360.1)	424.4 (18.2)	394.4 (25.6)
P values						
SOILTEMP	0.0901	** < 0.001**	** < 0.001**	0.8092	0.2200	0.2762
RAIN	0.1785	** < 0.001**	** < 0.001**	** < 0.001**	** < 0.001**	** < 0.001**
SOILTEMP*RAIN	0.6612	0.1884	0.1460	0.8542	0.6270	0.0982

Mean values for SOILTEMP (HEAT and CTRL, n = 6) and RAIN (DRY, MID, and WET, n = 4) with the standard error (in brackets). P values of two-way ANOVA for SOILTEMP*RAIN. The bold values indicate significance at p < 0.001

In contrast to spring barley during GP1, the sugar beet yield was higher under the influence of heat emission, at least under the LOESS treatment (5609.0 to 6608.0 g m^−2^). RAIN imposed a significant impact on the yield, with the highest values under WET, declining from MID to DRY.

There was no statistically significant difference between the CTRL and HEAT treatments for either soil type during GP3, although there was a slightly lower yield under the influence of heat emission. RAIN influenced the yield in a similar pattern to that of previous crops. There was no interaction effect between SOILTEMP and RAIN on the yield during the single growth phases.

In summary, significant differences were observed between HEAT and CTRL for yield and root growth, suggesting an important role for soil heat flux assessment in the field.

## Discussion

### Heat simulation

The soil water content near a heat source decreases due to the generated vapor flux [79]. The observed water condensation on the outside of the transparent tubes during root observation could be an indication of this phenomenon (Fig. 7). The soil water content is the main factor of the thermal conductivity, and the decrease in the soil water content leads to increased thermal resistivity [43, 80]. Heat emission was simulated by a temperature-controlled barrel heater (PID controller) instead of employing a general heat flux (W m^−1^). In our setup, this could indicate that the decline in the soil water content due to root growth caused an increase in the thermal resistivity. Hence, the temperature-controlled heater used less energy to maintain the preset temperature of 50 °C (reduced energy input) relative to the application of a constant input (steady energy input). This issue also pertains to the elevated temperatures in the greenhouses and could be an explanation for the smaller differences in the soil temperature between HEAT and CTRL at the higher ambient temperature (Fig. 4). For example, the average monthly energy consumption of one heater in August 2020 of 32 W/h versus 45 W/h in January supports this finding. Furthermore, this could influence the results for the interaction between the soil temperature and precipitation. It is recommended that the climate control of the greenhouses be improved, contingent upon the technical possibilities of the location.

In addition, the simulated day–night cycle during the first few weeks of the experiment relative to the later constant temperature of 50 °C could impact the growth of spring barley due to the circadian clock and may explain the greater effect on the yield during GP1 under HEAT [81, 82]. The renewed cultivation of spring barley at a constant temperature of 50 °C could improve our understanding of this phenomenon.

### Transferability to the field

In general, the cultivation of plants in greenhouses differs from that under field conditions in terms of various abiotic factors, as extensively discussed by Poorter et. al. [83]. Regarding the shoot environment in our experiment, the photosynthetically active light and UV-B radiation conditions differed from field conditions. Neither the wind speed nor air turbulence was present. The temperature was controlled to match the field conditions at the different BBCH stages, but under certain weather conditions (cloud-free, sunny and windy days), the vents were closed for safety reasons to prevent damage to the greenhouse. This led to temperature spikes to over 40 °C due to the weather conditions in late summer 2020 GP2 (Fig. 4). This might have caused additionally thermal stress for the plants above ground and therefore influenced the results. Conducting further experiments in climate-controlled greenhouses or under semioutdoor conditions could provide a solution to this problem.

To a greater extent, the root environment affects plant growth in greenhouses. The nutrient and water supply, soil compaction and temperature are related to the pot size. A correlation between the plant biomass and pot size was found by Poorter et al., with negative effects on the results if the pot size is too small [58]. We addressed this problem by building our HAL-M model with a sufficient soil volume and typical bulk density, thereby reducing the impact of an inadequate nutrition or water supply or influencing root growth while maintaining an inferior bulk density.

Among these crops, sugar beet has a high water demand, and during GP2, we investigated 17 plants per m^2^, which is a higher plant density than that in the field [84]. This approach was necessary because at least three biological replicates were needed per HAL-M to account for biological variability. More plants on less soil also indicated that more water was used per m^−2^, with less water available per individual plant, which led to a short-term water stress situation at the end of GP2. To specifically focus on the effect of heat emission, a water supply within the optimal range is reasonable, whereas precipitation under field conditions is often unfavorable.

### Comparison to natural soils

The soil temperature in the twelve control HAL-Ms depended on the average ambient temperature, and with increasing temperature, the soil temperature in the HAL-Ms accordingly increased. Compared with the mean soil temperature under field conditions at the 100-cm depth at three locations in Saxony-Anhalt, the soil temperature in the control HAL-M was 5–10 °C higher. This also affected the heated HAL-Ms.

The vertical temperature gradient that occurs in natural soils was not achievable with our setup. For barley, by managing a soil temperature gradient of 20 to 10 °C from top to bottom, shoot and root growth can significantly increase [85]. Hence, our uniform soil temperature in the control vessel was unfavorable. Considering this aspect, the difference between the heated and control HAL-Ms could vary under field conditions. Simulating this gradient in our setup would be challenging but would further improve the HAL-M model and enhance the results.

The surrounding ground, which functions as a thermal isolator under field conditions, was considered by applying an isolating material on the outside of the vessels (Armaflex XG 32). The thermal conductivity of soil is complex and is determined by the water content, bulk density, mineral component and porosity [43, 44]. Thus, this was only addressed on a basic level by preventing direct sunlight from heating the HAL-Ms.

Prior to the start of the experiment, we waterlogged the HAL-Ms until excess water was discharged from the bottom of the vessels, but we did not reach the calculated FC (Fig. 5). Considering that the FC concept remains contested from certain perspectives, our FC values were acceptable [86]. More importantly, during crop rotation, the starting values in some cases were not again reached, especially in the subsoil of the LOESS treatment. This was caused by maintaining the precipitation levels the same over the three simulated years, resulting in three consecutive DRY years that in turn caused an additional water deficit during GP2 and GP3, mainly under the LOESS treatment. Compared to the regional precipitation in recent years, this precipitation pattern is not uncommon and reflects the predicted climatic challenges in the future [87]. However, refilling the HAL-Ms to the field capacity before the start of each new GP could yield another outcome.

Overall, this influences the transferability and generality of our results to the field. If the focus of the research is to move away from the plant and more toward soil function, it would make sense to measure the entire water retention curve, add more probes at additional depths, and calibrate them accurately.

### Root observation

Minirhizotron tubes were installed during HAL-M construction. We carefully filled and compacted the soil around the tubes, leading to a tight soil connection without scratching the surface of the tubes, which can usually occur during installation in the field. In some experiments, minirhizotron setups were installed with a slope angle less than 45°, which aims to prevent preferential root growth [57]. However, we used an angle of 45° and installed both tubes in an offset manner. This approach increased the observation area per unit and reduced interference. Problems with preferential root growth due to the 45° angle were not observed. The three levels of precipitation did not affect the root growth observed in our setup. However, Boudiar et al. showed for different barley cultivars that plants may tend to produce more root biomass under drought stress [88]. More detailed studies on root growth could be made by using x-ray computed tomography technology to examine the root system directly in the HAL-Ms [89, 90].

### Possible interactions between HEAT, root growth and soil water content

Root growth is a key component of plant growth and yield [91]. Experiments with barley showed that a larger root system positively affects the yield [92]. However, root growth can be affected by various conditions. Temperature is one of the main factors, which has been known since the late nineteenth century under the term thermotropism [93]. Higher temperatures, such as those resulting from the simulated heat emission conditions in our experiment, could affect root growth in different ways and at different intensities [48, 49]. The lower intensity of roots under HEAT during GP1 could therefore be attributed to heat emission (negative influence). As a result, the usable soil water in the heated HAL-Ms was not exhausted during GP1 (Fig. 5), and sugar beet during GP2 could use this additional water due to its unaffected root growth. Although root intensity in GP3 was similarly reduced due to HEAT as in GP1, there was no significant difference in soil water content between the HEAT and CTRL treatments. Notably, the root architecture and hydraulics can determine the water uptake capacity of plants [94]. Moreover, HEAT may have affected these parameters for spring barley during GP1 but not for spring wheat during GP3, although the root intensity was reduced.

## Conclusions

The experimental setup was designed to specifically examine potential heat flow effects in Saxony-Anhalt resulting from the planned SuedOstLink, a 525-kV HVDC underground power line traversing this state. However, it can also be used for a wide variety of experiments.

We have designed and constructed low-cost large HeAted soiL Monoliths (HAL-Ms) for simulating heat flows in soil, with a natural soil composition and density. It is of significant importance to consider the construction of standardized experimental pots, such as those proposed here, in the context of the planned underground power lines that are to be installed worldwide. This is particularly relevant in light of the lack of clarity regarding the potential environmental impact of such installations. We observed root growth, temperature and water content over an extended period. Nevertheless, the HAL-Ms are sufficiently flexible to accommodate additional research questions and the observation of additional parameters.

We performed a field trial-type experiment involving complete crop rotation in a greenhouse. All the crop plants were grown to maturity and produced acceptable yields compared to those under field conditions.

Moreover, we showed that under the simulated conditions, heat emission reduces the yield of spring barley. Root growth of spring barley and spring wheat was significantly affected near the heat source. The underlying physiological mechanism remains unknown and should be further investigated. The differing outcomes for the crops and the two investigated soils indicate that the regional effect of heat emission and crop specific differences could lead to diverse results. Thus, further investigations of upcoming heat-emitting underground cables with regionally affected soil compositions and crops are necessary to evaluate the impacts of these cables on food production and economic aspects of farmers. Moreover, this experimental design could serve as a low-cost, fast and reliable standard for investigating thermal issues related to various soil compositions and types, precipitation regimes and crop plants affected by future projects.

The design is expandable to soil biota and soil plant interactions that are potentially vulnerable to artificial heat emission in soil.

In the unheated mode, HAL-Ms could also serve to uncover the mechanisms of plant–soil feedbacks under various abiotic and biotic drivers [95] by conducting root phenotyping in natural soils [96] and evaluating the use of biologicals, e.g., endophytes, which are not domestic in Europe and therefore not easy to use in field experiments due to legal regulations [97] or for performing drought experiments, mostly involving the use of inappropriate pots [98].

The HAL-M model could thus serve as a link between pot and field trials. With the advantages of both methods, they enrich many research areas with the aim of controlling natural soil and plant conditions.

## Data Availability

The authors do not have permission to share data.
